# HfS_2_/MoTe_2_ vdW heterostructure: bandstructure and strain engineering based on first-principles calculation

**DOI:** 10.1039/c9ra10087c

**Published:** 2020-01-15

**Authors:** Xinge Yang, Xiande Qin, Junxuan Luo, Nadeem Abbas, Jiaoning Tang, Yu Li, Kunming Gu

**Affiliations:** Shenzhen Key Laboratory of Advanced Functional Material, College of Material Science and Engineering, Shenzhen University Shenzhen Guangdong 518060 China kmgu@szu.edu.cn liyu@szu.edu.cn; Key Laboratory of Optoelectronic Devices and Systems of Ministry of Education and Guangdong Province, College of Optoelectronic Engineering, Shenzhen University Shenzhen Guangdong 518060 China

## Abstract

In this study, a multilayered van der Waals (vdW) heterostructure, HfS_2_/MoTe_2_, was modeled and simulated using density functional theory (DFT). It was found that the multilayers (up to 7 layers) are typical indirect bandgap semiconductors with an indirect band gap varying from 0.35 eV to 0.51 eV. The maximum energy value of the valence band (VBM) and the minimum energy value of the conduction band (CBM) of the heterostructure were found to be dominated by the MoTe_2_ layer and the HfS_2_ layer, respectively, characterized as type-II band alignment, leading to potential photovoltaic applications. Optical spectra analysis also revealed that the materials have strong absorption coefficients in the visible and ultraviolet regions, which can be used in the detection of visible and ultraviolet light. Under an external strain perpendicular to the layer plane, the heterostructure exhibits a general transition from semiconductor to metal at a critical interlayer-distance of 2.54 Å. The carrier effective mass and optical properties of the heterostructures can also be modulated under external strain, indicating a good piezoelectric effect in the heterostructure.

## Abstract 2

In this study, a multilayered van der Waals (vdW) heterostructure, HfS_2_/MoTe_2_, was modeled and simulated using density functional theory (DFT).

## Graphical abstract

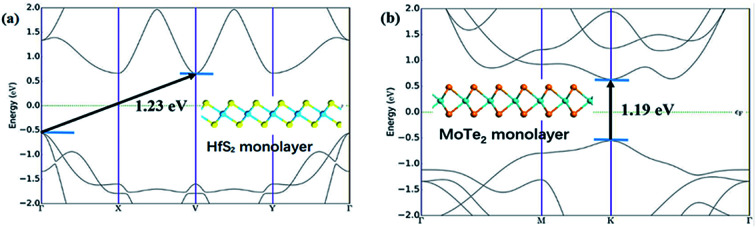

## 1 Introduction

In the past decade, two-dimensional materials (2D materials) have attracted great interest due to their inherent ultra-thin features and robust lattice structure.^1–3^ Among them, the family of transition metal disulfide (TMD) materials, a new generation of optoelectronic functional materials with rich physical properties, known as post-graphene materials have wide application prospects.^4^ The unique optical properties of TMDs are considered to have superior potential in the field of micro/nanoelectronics applications to graphene, which lacks an intrinsic band gap.^5–7^ Similar to graphene, monolayer and multi-layer TMD sheets can be obtained using classical mechanical exfoliation or chemical approaches.^8^ Since the first report of single-layered MoS_2_ (ref. 9) various groups worldwide have worked extensively on the properties of TMD materials and the characteristics of TMD devices. Recently, TMDs such as MoS_2_,^2,8^ MoSe_2_,^10^ MoTe_2_,^11–13^ WS_2_,^14^ WSe_2_ ^15^ and so on have been the focus of a lot of research.

Hf-based TMDs (such as HfSe_2_ and HfS_2_) have been discovered as a new type of TMDs semiconductor with small band gap, large work function, high mobility and so on.^16^ In recent years, several nanosheets with layered structure have been successfully prepared by chemical vapor deposition (CVD).^17^ Similar to MoS_2_ and other widely studied TMDs, HfS_2_ is also a layered semiconductor. Recent studies showed that single-layer HfS_2_ is an indirect bandgap semiconductor with an octahedral coordinate structure, which is different from MoS_2_.^18,19^ Because of their short response time, high photosensitivity and good field effect response, they have shown bright prospects in the field of photodetectors^17,20^ and field effect transistors (FETs).^21^ In addition, theoretical calculations showed that 2H-HfS_2_ and 1T-HfS_2_ monolayers have potential applications in water splitting as photocatalysts^22,23^ and optical devices.^24,25^

Combining two different semiconductor materials to form heterostructures and obtain different properties is an important approach for semiconductor materials research.^26^ Functional materials based on heterostructures are the cornerstone of many applications, such as semiconductor lasers and light-emitting diodes.^27,28^ For TMDs, researchers can also arrange different TMD materials into heterostructures, including parallel heterostructures and vertical heterostructures, of which the vertical stacking of 2D vdW heterostructures attracts more attention.^29–31^ vdW heterostructures provide an alternative method for low energy material integration, by which selected materials are physically assembled through weak vdW interactions.^32^ This physical assembly method does not depend on one-to-one chemical bonds,^33^ nor does it involve direct chemical treatment of existing materials, nor is it limited to materials with similar lattice structure or compatible synthesis conditions.^32–35^ Therefore, it has aroused great interest, making different 2D atomic crystals have highly different lattice structures at the interface, but almost no chemical disorder. At the same time, it provides more operational possibilities for the application of device technology, resulting in various novel physical phenomena, such as integer and quantum molecular Hall effect,^36,37^ quantum spin Hall effect^38^ and exciton–polaron condensation.^39^ Both theoretical and experimental results showed that vdW heterostructures can possess good optical and electrical properties, showing potential applications in the next-generation electronic devices.^40–42^

Applying strain onto vdW heterostructures is also an effective way to moderate their band structure and other physical properties, which can be achieved by stretching or bending an elastic substrate with an applied stress. For example, strain can also reduce photoluminescence and regulate polarization associated with direct or indirect band gap transitions.^43^ There have been many reports on the simulation and modeling of strain-activated band structure conversion. The effects of strain on electrical, optical, and magnetic properties have also been well studied and predicted.^44,45^

HfS_2_ has high electron affinity, which results in a large band discontinuity when heterostructures are formed with other TMDs such as MoS_2_.^46,47^ However, up to now, little research has been done on vdW heterostructures based on HfS_2_ and MoX_2_ (X = S, Se, Te).^46,47^ Considering that the lattice mismatch between HfS_2_ and MoTe_2_ is the smallest under the same conditions when the heterostructure is constructed, in this work, the HfS_2_/MoTe_2_ vdW heterostructure was modeled and simulated by means of DFT method. The band structure, effective mass, electron transport and total energy of the structure were investigated with respect to the number of layers and a positive compression strain. It was found that the HfS_2_/MoTe_2_ heterostructure shows type II band alignment and its band gap varies with the vertical strains, which indicates that the photoelectric excitation effect and good piezoelectric effect can be achieved in the HfS_2_/MoTe_2_ heterostructures.

## 2 Method

When constructing multi-layer heterostructures, HfS_2_ and MoTe_2_ surfaces of 1 × 1 unit cell were coupled to minimize the lattice mismatch between stacked monolayers. In this study, the band structure, effective mass and tensile and compressive strains of 2D HfS_2_/MoTe_2_ vdW heterostructures (bilayer-to-multilayer films) were studied by comprehensive DFT method using the Virtual Nanolab Atomistix ToolKit (VNL-ATK) software package. The general gradient approximation (GGA) with Perdew–Burke–Ernzerhof (PBE) exchange correlation function was used for geometric optimization and band structure calculation without considering spin–orbit coupling. The Brillouin region was represented by Monkhorst_Pack's special *k*-point grid (5 × 5 × 1 for geometric optimization and 15 × 15 × 1 for electronic structure calculation). The basis set was set to SG15-medium with a mesh cut-off energy of 85 Ha. The PulayMixer algorithm was employed as iteration control parameter with tolerance value of 10^−4^ Ha. The force tolerance was set to 0.01 eV·Å^−1^ and strain tolerance was set to 0.001 eV·Å^−3^ for geometric optimization. Moreover, in order to avoid the interaction between adjacent layers in periodic plates, a vacuum layer larger than 15 Å was added. To evaluate the influence of the distance between layers, the geometry of multilayers were optimized under the constraint of a fixed distance *d* between layers. All calculations were ensured convergence.

## 3 Results and discussion

### 3.1 Structural and electronic properties

**Fig. 1:**
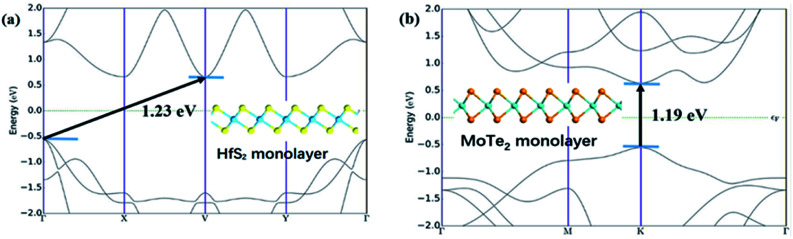
(a) Band structure of monolayer HfS_2_. The illustration is the side view of HfS_2_. (b) Band structure of monolayer MoTe_2_. The illustration is the side view of MoTe_2_.

Fig. 1 shows the lattice structure and energy band structure of 1T-HfS_2_ and 2H-MoTe_2_ monolayer, respectively. HfS_2_ adopts triangular and layered CdI_2_ structure (space group *P*3̄*m*1). In the *ab* plane (intra-layer), the chemical bonds between metal and sulfur atoms are hybrid ion/covalent bonds, while the inter-layer is superimposed by the weak vdW interaction along the *c* axis (cross-plane).^48,49^ Each metal atom is surrounded by six sulfur atoms in a triangular prism, and each S atom is connected to three metal atoms. The fact that HfS_2_ possesses a structure similar to CdI_2_ makes it possible to systematically study their cyclical and group trends.^50,51^ With different stacking sequences, MoTe_2_ presents different phase structures (2H, 1T, 1T′) and the 2H phase with space group *P*6_3_/*mmc* is the most stable one at room temperature. Each Mo atom is surrounded by six Te atoms in the prism.^52^ The optimized lattice constant of HfS_2_ monolayer (1T) is *a* = *b* = 3.64 Å, showing nonmagnetic semiconductor characteristics with an indirect energy bandgap of 1.23 eV. The calculated lattice constant of MoTe_2_ monolayer (2H) is *a* = *b* = 3.56 Å. It is also a nonmagnetic semiconductor with a direct energy bandgap of 1.19 eV. These results in our simulation are well consistent with experimental and other DFT values.^5,27,28^

**Fig. 2:**
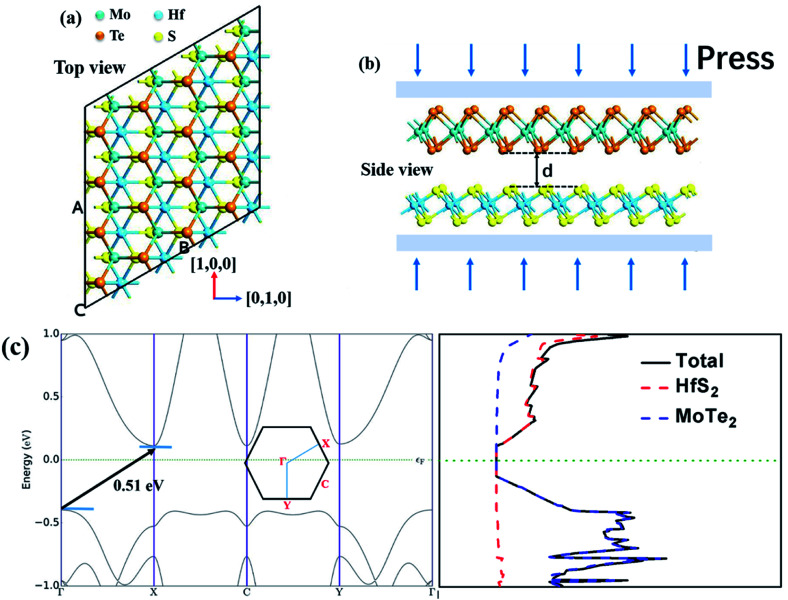
The HfS_2_/MoTe_2_ bilayer heterostructure. Yellow ball and light blue ball represent sulfur and hafnium, respectively. Orange and dark blue balls represent tellurium and molybdenum, respectively. (a) Top view: [1, 0, 0] represents zigzag direction, [0, 1, 0] represents armchair direction. (b) Side view: *d* representation layer distance. (c) Band structure and DOS of HfS_2_/MoTe_2_ bilayer heterostructure. The illustration is a Brillouin region with a high symmetry label.

The optimized geometry of HfS_2_/MoTe_2_ vdW heterostructure is shown in Fig. 2(a) and (b). It consists of a HfS_2_ monolayer and a MoTe_2_ monolayer, with a minimum lattice mismatch of about 2.37% between the two unit cells. Fig. 2(c) is the energy band structure of the HfS_2_/MoTe_2_ bi-layer, showing the calculation along the Brillouin zone path marked with high symmetry points. It exhibits the characteristics of an indirect energy bandgap semiconductor, which is similar to the previously studied heterogeneous bilayer.^53^ From the band structure and the electronic density of states (DOS), the VBM is mainly the contribution of the MoTe_2_ layer, while the CBM is due to the HfS_2_ layer.

**Fig. 3:**
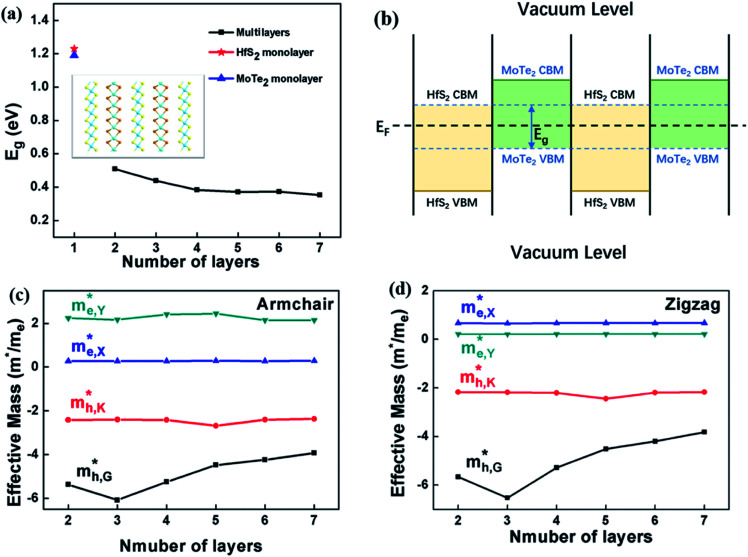
(a) The bandgap of HfS_2_/MoTe_2_ multilayer heterostructure varies with respect to the number of layers. The illustration is a HfS_2_/MoTe_2_ multilayer heterostructure. (b) Schematic illustration of *E*_g_ after realignment of Fermi levels. (c) and (d) The effective mass of electrons and holes along the armchair and zigzag directions varies with the number of layers, respectively. Where 
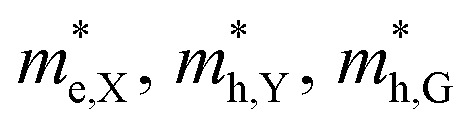
 and 
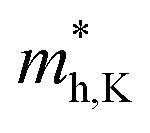
 are the electron effective mass in the conduction band at point X and point Y, and the hole effective mass in the valence band at point G and point K [0.5, 0.385, 0], respectively.

Multilayer heterostructures consisting of alternately stacked HfS_2_ and MoTe_2_ layers remain indirect bandgap semiconductors when the number of layer increases up to 7. Fig. 3(a) demonstrates the energy gap value (*E*_g_) of the multilayer heterostructure as a function of the number of layer. All of them are indirect bandgap semiconductors with *E*_g_ decreasing from 0.51 eV to 0.35 eV, which does not change much with the number of stacking layer. The *E*_g_ of the multilayer heterostructure is much lower than that of HfS_2_ and MoTe_2_ monolayer, which probably due to the rearrangement of Fermi levels between adjacent layers. Fig. 3(b) shows a schematic view of the band alignment of the alternatively stacked HfS_2_/MoTe_2_ multilayer heterostructure. There is significant difference in the electron affinity between MoTe_2_ layer and HfS_2_ layer, leading to rearrangement mechanism of band structure.^54^ As to the band structure of the heterogeneous multilayer, it was also found that the CBM is attributed to the HfS_2_ monolayer and the VBM is due to the MoTe_2_ monolayer. As the number of layers increases, the bandgap value changes slightly.

In addition to energy band gap, the effective mass at VBM and CBM are also important parameters for semiconductors. The in-plane effective mass is the most interesting issue because it is related to the in-plane carrier mobility of such 2D semiconductors. The effective mass of electrons and holes m* can be obtained by using eqn (1):1
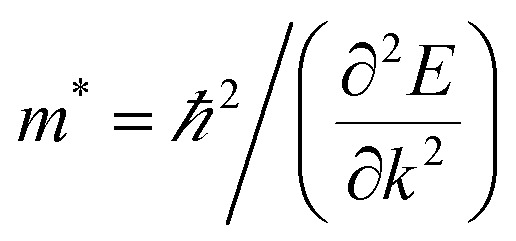


According to the energy band structure, the electron effective mass at the conduction band edge at point Y (0, 0.5, 0), and the hole effective mass at the valence band edge at point K (0.5, 0.385, 0) and point G (Γ) (0, 0, 0), labeled as 
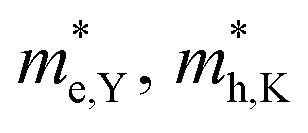
 and 
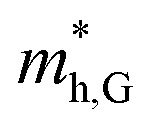
, respectively, were calculated. For the bilayer heterostructure, CBM is located at point Y and VBM is located at point G. However, with increasing of stacking layer, VBM moves from point G to point K. Fig. 3(c) and (d) illustrates that the effective mass of HfS_2_/MoTe_2_ heterogeneous multilayers is also changed with the number of layer. Whether for the direction along zigzag or armchair direction, the change of the effective mass with respect to the number of layer is almost the same. The effective mass of 
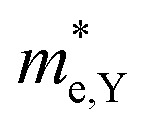
 and 
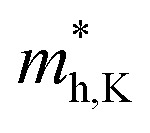
 do not change obviously with the number of layers, but 
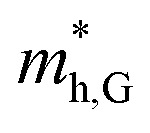
 does. For the VBM of the multilayers, it's located at point G (Γ) for the bilayer and and 5- to 7-layer structures. But for the 3-layer and 4-layer structure, it's located at point K, leading to a larger 
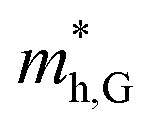
 for the 3-layer and 4-layers.

The engineering of 2D materials energy bandgap, whether in theory or in experiment, is always a hot spot of research. For layered materials interacted by interlaminar vdW forces, the interlaminar distance will be affected when applying an external force perpendicular to the plane. Here, the effect of applying vertical strain (tensile strain and compressive strain, shown in Fig. 2(b)) on the band structure and effective mass of the HfS_2_/MoTe_2_ bilayer was carried out.

**Fig. 4:**
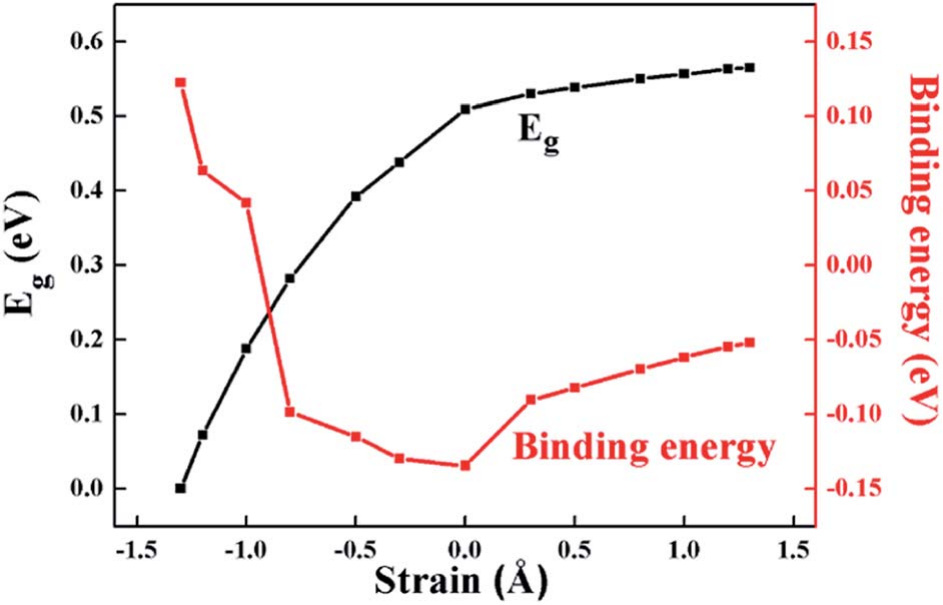
Binding energy and band gap of HfS_2_/MoTe_2_ bilayer heterostructure varies with strain value.

**Fig. 5:**
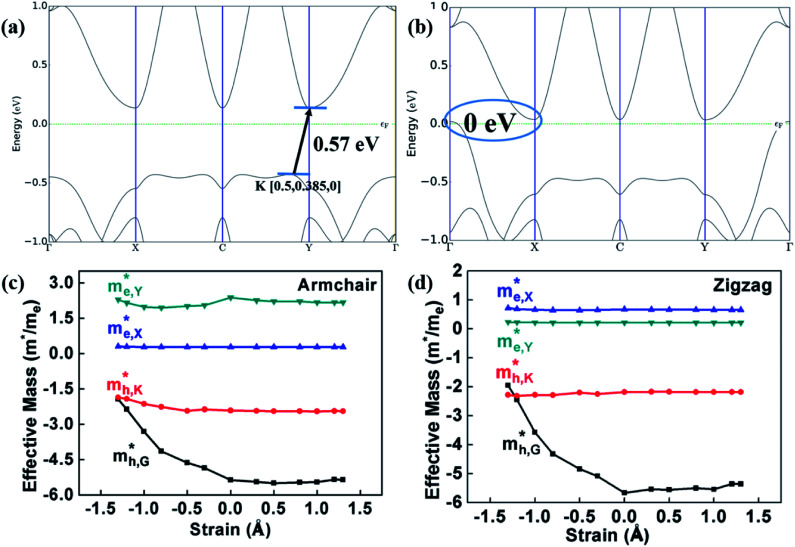
(a) and (b) Band structure with tensile strain of Δ*d* = 1.3 Å and compressive strain of Δ*d* = 1.3 Å, respectively. (c) and (d) The effective mass of electrons and holes along the armchair and zigzag directions of HfS_2_/MoTe_2_ bilayer heterostructure varies with strain value.

According to Fig. 4 and 5(a) and (b), from the equilibrium state, it is expected that in the process of applying compressive strain (or tensile strain), as the interlayer distance *d* deviates from the equilibrium state, the binding energy of the heterostructure will increase. Moreover, it was revealed that the HfS_2_/MoTe_2_ bilayer undergoes a phase transition from semiconductor to metal during compressive strain: the crucial interlayer distance *d* is 2.54 Å. Similar phenomena were reported in BP/MoS_2_ heterostructure.^29^ It proves that HfS_2_/MoTe_2_ vdW heterostructure is a compression controllable material that can be used to coordinate the bandgap and has broad application prospects in nanoelectronics and optoelectronics. In the process of tensile strain, the value of the energy bandgap increases from 0.51 eV to 0.56 eV. The VBM shifts from point G to point K and the CBM shifts from point X to point Y. Meanwhile, under external strain, regardless of compressive strain or tensile strain, it is obvious that the CBM and the VBM are still dominated by the HfS_2_ layer and the MoTe_2_ layer, respectively. The stable type-II energy band alignment characteristics makes it possible for spatially separating the electrons and holes in the HfS_2_ layer and the MoTe_2_ layer, respectively. The results exhibit that the HfS_2_/MoTe_2_ vdW heterostructure may be suitable for optoelectronic solar energy conversion.

Fig. 5(c) and (d) demonstrate that, under compressive strain or tensile strain, the variation of the effective mass of the HfS_2_/MoTe_2_ vdW heterostructure is similar in both zigzag and armchair directions. Among them, 
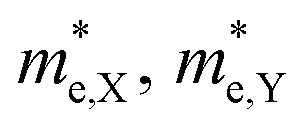
 and 
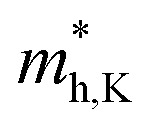
 are almost constant, whereas 
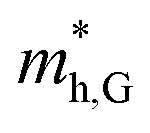
 decreases rapidly with decreasing *d*. It can also be seen that the minimum values of 
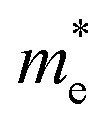
 is much smaller than 1, indicating high mobility and conductivity.^29^

In order to better understand the charge transfer between HfS_2_ and MoTe_2_ monolayer in HfS_2_/MoTe_2_ vdW heterostructure system, the differential charge density of it was calculated. The formulas for calculating the three-dimensional electron difference density Δ*n* are as follows:

2 Δ*n* = *n*_HfS_2_/MoTe_2__ − *n*_HfS_2__ − *n*_MoTe_2__

where *n*_HfS_2_/MoTe_2__, *n*_HfS_2__ and *n*_MoTe_2__ represent the charge density of HfS_2_/MoTe_2_ vdW heterostructure, HfS_2_ monolayer and MoTe_2_ monolayer, respectively. In addition, the plane average electron density Δ*n*_r_ is calculated by the integrated electron density difference method along the *x*–*y* plane, and the formula is as follows:3
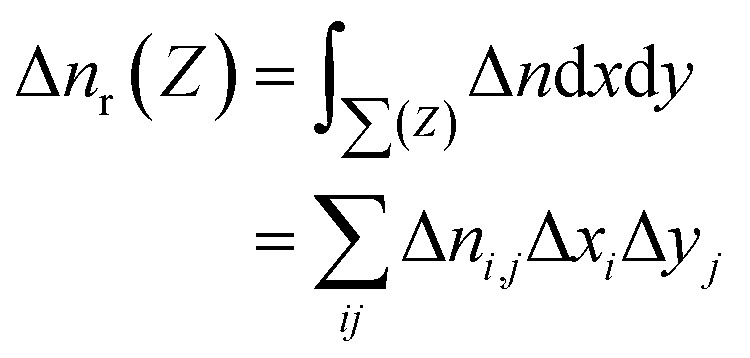
where the *z*-axis is the direction normal to the interface, and the *x*–*y* plane is the super-section perpendicular to the *z*-axis; *i* and *j* represent the *a*-axis and the *b*-axis, respectively.

**Fig. 6:**
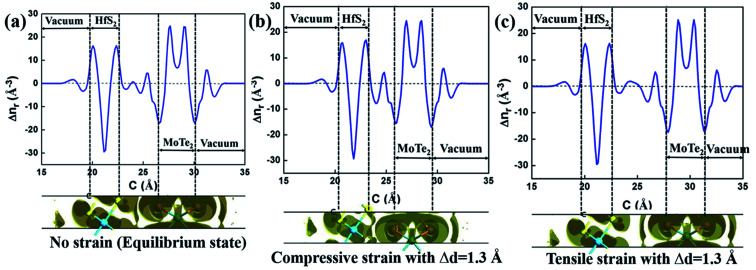
The electron difference density under different strains of HfS_2_/MoTe_2_ bilayer heterostructure. The meaning of distance here is the increment of d value. (a) No strain (equilibrium state). (b) Compressive strain with Δ*d* = 1.3 Å. (c) Tensile strain with Δ*d* = 1.3 Å.

Fig. 6 exhibit the Δ*n* and Δ*n*_r_ of HfS_2_/MoTe_2_ heterostructures under different vertical strain states. The Δ*n* at the bottom of Fig. 6(a) indicates that the charge redistribution occurs at the interface and inside the material in the equilibrium state. The Δ*n*_r_ display at the top of Fig. 6(a) shows that part of the charge depletion mainly occurs in the middle of the interface and on the surface of HfS_2_ and MoTe_2_. The charge accumulation is concentrated near MoTe_2_ at the interface. This indicates that more photogenerated carriers can be shared between HfS_2_ and MoTe_2_ after photoexcitation.^55^ As shown at the bottom of Fig. 6(b), charge accumulation occurs mainly in the middle of the interface, while charge depletion occurs on the surface of HfS_2_ and MoTe_2_ when it becomes metallic under compressive strain. This suggests that some conduction bands and valence bands in HfS_2_/MoTe_2_ depend on both HfS_2_ and MoTe_2_. In heterostructure, the wave function mixing in HfS_2_/MoTe_2_ is higher with the enhancement of interlayer interaction.^56^ In the tensile strain process of Fig. 6(c), charge depletion occurs mainly in the middle of the interface and on the surfaces of HfS_2_ and MoTe_2_, while charge accumulation occurs near the charge depletion in the middle of the interface.

### 3.2 Optical properties

**Fig. 7:**
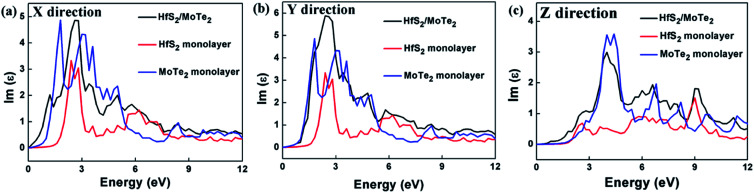
Dielectric constant (image part *ε*_2_) of HfS_2_ monolayer, MoTe_2_ monolayer and HfS_2_/MoTe_2_ bilayer heterostructure. (a) *X* direction. (b) *Y* direction. (c) *Z* direction.

Since materials with type-II heterostructure alignment are regarded to make hole–electron separation more efficient, and can be used for optical absorption in a wider range of solar spectra,^53,57^ in order to reveal the unique characteristics of HfS_2_/MoTe_2_, a detailed discussion of its imaginary part of the dielectric constant *ε*_2_ for light absorption is given. In the linear response range, the complex dielectric response function to describe the optical properties of a material is *ε* = *ε*_1_ + i*ε*_2_, where *ε*_1_ denotes the real part calculated by the well-known Kramers–Kronig relation and *ε*_2_ is the imaginary part obtained from the matrix elements of the wave function for occupied and non-occupied states. At the same time, the *ε*_2_ can also be used to evaluate the light absorption properties of the material.^51^ Unlike the hexagonal lattice and cubic symmetry, the dielectric constant of HfS_2_/MoTe_2_ is anisotropic. Three different dielectric tensors can be observed, *ε*^*xx*^, *ε*^*yy*^, and *ε*^*zz*^. The dielectric constants of HfS_2_/MoTe_2_ heterostructures, HfS_2_ monolayers and MoTe_2_ monolayers are illustrated in Fig. 7. Strong anisotropy is observed in the imaginary parts of the dielectric function *ε*_2.5_^*xx*^, *ε*_2.5_^*yy*^ and *ε*_4_^*zz*^. They all are the macroscopic phenomena of electron transitions between energy bands in solid under the perturbation of photoelectric magnetic field.^57^ Obviously, at both the *X* and *Y* directions the dielectric peaks are in the visible range, while at the *Z* direction the dielectric peak is in the ultraviolet region.

**Fig. 8:**
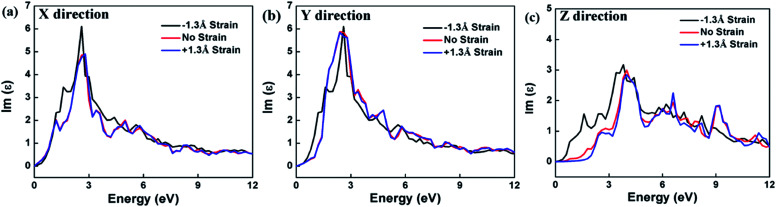
Dielectric constant (image part *ε*_2_) of HfS_2_/MoTe_2_ multilayer heterostructure. (a) *X* direction. (b) *Y* direction. (c) *Z* direction.

**Fig. 9:**
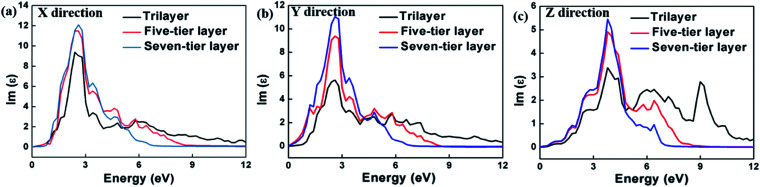
Dielectric constant (image part *ε*_2_) of HfS_2_/MoTe_2_ bilayer heterostructure under different strains. (a) *X* direction. (b) *Y* direction. (c) *Z* direction.

The calculation of the imaginary part of the dielectric constant *ε*_2_ of the HfS_2_/MoTe_2_ multilayer heterostructure as a function of energy is illustrated in Fig. 8. The *X*, *Y* and *Z* directions of the multilayers are consistent with those of the bilayer heterostructure, which indicates that the multilayers have good applications in the detection of ultraviolet (above 3.11 ev) and visible light (1.61–3.11 ev). Fig. 9 shows the influence of strain on *ε*_2_ of the HfS_2_/MoTe_2_ bilayer. It can be seen that, in the *X* and *Y* directions, no matter for tensile strain or compressive strain, the strain effect on *ε*_2_ is not obvious. However, in the *Z* direction, *ε*_2_ is affected significantly by external strain. The anisotropic strain dependency is attributed to the direction of strain. In our work, strains along *Z*-direction only affects the interlayer interaction and has little effect on the intralayer interaction.

## 4 Conclusion

In summary, first principle calculation method was used to explore in detail the construction and bandstructure, effective mass and strain effect of the hybrid layered HfS_2_/MoTe_2_ vdW heterostructure. It was found that the heterostructures are typical type-II semiconductor with an indirect band gap from 0.35 eV to 0.51 eV, in which the VBM is provided by HfS_2_ layer and the CBM is provided by MoTe_2_ layer. The type-II semiconductor feature is stable in multilayered heterostructure with the number of layer up to 7, which makes it a good candidate for applications in solar photoelectric conversion. In the process of applying vertical strain to the heterostructure, it is found that the energy band gap decreases with compressive strain and a transition from indirect semiconductor to metallic phase was found at a crucial interlayer distance *d* of 2.54 Å, indicating a band-coordinated structure that can be used in sensing components. The optical absorption range of bilayer-heterostructure is in the visible and ultraviolet range. In the meantime, during the vertical strain process, the optical properties have slight effect in the *X* and *Y* directions, though only have a significant effect along the vertical direction of compression, indicating that the HfS_2_/MoTe_2_ vdW heterostructure can be used not only for visible light and ultraviolet optical detection elements. It can also have broad application prospects in optoelectronic devices such as photovoltaic cell logic devices in the future.

## Conflicts of interest

There are no conflicts to declare.

## Supplementary Material
